# Global Profile of tRNA-Derived Small RNAs in Pathological Cardiac Hypertrophy Plasma and Identification of tRF-21-NB8PLML3E as a New Hypertrophy Marker

**DOI:** 10.3390/diagnostics13122065

**Published:** 2023-06-14

**Authors:** Jingyi Xu, Buyun Qian, Feng Wang, Ying Huang, Xinxin Yan, Ping Li, Qian Zhang, Yuan Li, Kangyun Sun

**Affiliations:** 1 Department of Cardiology, The Affiliated Suzhou Hospital of Nanjing Medical University, Gusu School, Nanjing Medical University, Suzhou 215008, China; xujingyigs@126.com (J.X.); buyuncc712@163.com (B.Q.); liyuan1596215@163.com (Y.L.); 2 Department of Central Laboratory, The Affiliated Suzhou Hospital of Nanjing Medical University, Gusu School, Nanjing Medical University, Suzhou 215008, China; yinghuangsz@hotmail.com (Y.H.); liping121200@126.com (P.L.); 3 Department of Pharmacy, The Affiliated Suzhou Hospital of Nanjing Medical University, Gusu School, Nanjing Medical University, Suzhou 215008, China; wangfeng@alu.suda.edu.cn (F.W.); yxinxin2221@163.com (X.Y.); szsyyjk@163.com (Q.Z.)

**Keywords:** transfer RNA-derived small RNAs, pathological cardiac hypertrophy, small RNA sequencing, biomarker

## Abstract

Background: It remains unclear whether transfer RNA-derived small RNAs (tsRNAs) play a role in pathological cardiac hypertrophy (PCH). We aimed to clarify the expression profile of tsRNAs and disclose their relationship with the clinical phenotype of PCH and the putative role. Methods: Small RNA sequencing was performed on the plasma of PCH patients and healthy volunteers. In the larger sample size and angiotensin II (Ang II)-stimulated H9c2 cells, the data were validated by real-time qPCR. Atrial natriuretic peptide (ANP) and brain natriuretic peptide (BNP) were examined in Ang II-stimulated H9c2 cells. The potential role of tsRNAs in the pathogenesis of PCH was explored by bioinformatics analysis. Results: A total of 4185 differentially expressed tsRNAs were identified, of which four and five tsRNAs were observed to be significantly upregulated and downregulated, respectively. Of the five downregulated tsRNAs, four were verified to be significantly downregulated in the larger sample group, including tRF-30-3JVIJMRPFQ5D, tRF-16-R29P4PE, tRF-21-NB8PLML3E, and tRF-21-SWRYVMMV0, and the AUC values for diagnosis of concentric hypertrophy were 0.7893, 0.7825, 0.8475, and 0.8825, respectively. The four downregulated tsRNAs were negatively correlated with the left ventricular posterior wall dimensions in PCH patients (r = −0.4227; r = −0.4517; r = −0.5567; r = −0.4223). The levels of ANP and BNP, as well as cell size, were decreased in Ang II–stimulated H9c2 cells with 21-NB8PLML3E mimic transfection. Bioinformatics analysis revealed that the target genes of tRF-21-NB8PLML3E were mainly enriched in the metabolic pathway and involved in the regulation of ribosomes. Conclusions: The plasma tRF-21-NB8PLML3E might be considered as a biomarker and offers early screening potential in patients with PCH.

## 1. Introduction

**Figure 1:**
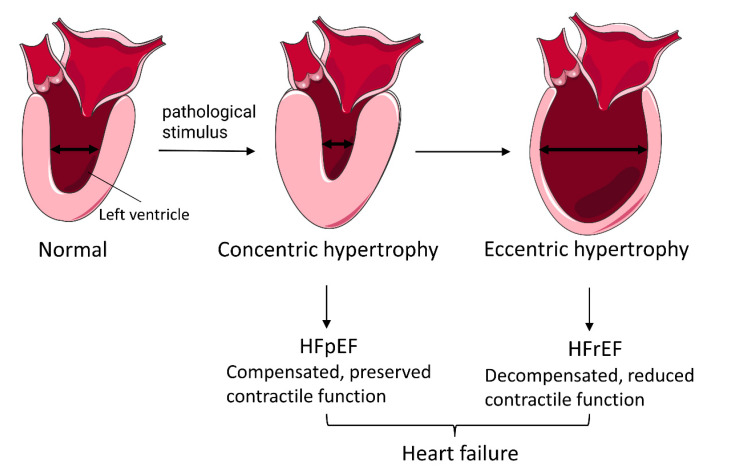
Overview of pathological cardiomyocyte hypertrophy. Pathological hypertrophy is initially identified by a reduction in ventricular-chamber dimension with increased wall thickness (concentric hypertrophy), which results in HFpEF. As the disease progresses, pathological hypertrophy leads to ventricular-chamber dilatation (eccentric hypertrophy) with impaired contractile function (maladaptive remodeling) which often results in HFrEF.

Cardiac hypertrophy is a physiological adaptive response of the heart to maintain cardiac function against physiological or pathological cardiac overloading that is characterized by an increased cardiomyocyte size and heart mass [1]. However, sustained pathological stress can lead to pathological cardiac hypertrophy (PCH), which manifests as enlarged cardiomyocyte size, cardiac fibrosis, and cardiomyocyte death [1,2]. As the disease progresses, pathological hypertrophy changes from the reduction in ventricular chamber dimension with increased wall thickness to the ventricular chamber dilatation in the later stages, progressively leading to heart failure (Figure 1) [3]. A better understanding of the key molecules in PCH may allow early diagnosis and targeted therapy.

Previous studies have shown that noncoding RNAs (ncRNAs) play a significant role in the occurrence and development of PCH via the regulation of cellular metabolism, translation regulation, and epigenetic modification [4,5,6]. Transfer RNAs (tRNAs) are a class of ncRNAs that delivers specific amino acids to ribosomes for protein synthesis [7]. It has been demonstrated that tRNAs can produce small ncRNAs, known as tRNA-derived small RNAs (tsRNAs) [8,9]. In terms of the cleavage positions of tRNA precursors or mature transcripts, tsRNAs can be divided into two types, namely, tRNA-related fragments (tRFs) and tRNA halves (tiRNAs) [10]. Based on their mapped positions, tsRNAs can be divided into the following five categories: tRF-1, tRF-3, tRF-5, internal tRF (i-tRF), and tiRNAs [9,11,12].

Many studies have demonstrated that tsRNAs are not simply random products of tRNA degradation, although their specific biological functions have not been fully clarified [13,14]. They are involved in regulating various stages of gene expression, including transcription, translation, RNA processing, and maturation, and are related to key cellular processes such as self-renewal, differentiation, and proliferation [15,16,17]. For example, it has been demonstrated that tsRNAs can regulate mRNA stability in an RNA interference manner or regulate protein translation via competitive binding to translation initiation complexes YBX1 [18,19].

As the most ancient small RNAs in all domains of life, tsRNAs have good sequence conservation [20]. Furthermore, tsRNAs are present in all types of body fluids [21] and are dynamically regulated in pathophysiological conditions [22]. Based on these properties, tsRNAs might be excellent candidates for liquid biopsy biomarkers.

Due to sequence conservation, tsRNAs play a variety of functions in fundamental biological processes, including gene silencing, ribosomal biogenesis, retro transposition, and epigenetic inheritance [20]. These functions make it possible for tsRNAs to regulate gene expression at the transcriptional and post-transcriptional levels, thereby playing a key regulatory role in multiple pathological events [23]. However, a full understanding of the tsRNAs’ potential role in PCH remains elusive.

In recent years, the application of tsRNAs in cardiovascular diseases has received growing attention. It has been shown that 5’-tiRNA-Cys-GCA can inhibit aortic dissection via the signal transducer and activator of the transcription 4 signaling pathway [24]. The synthetic tRF (GlnCTG) can negatively regulate the expression of FAS cell surface death receptors to elevate the proliferation and migration of vascular smooth muscle cells [25]. tiRNA-Gln-TTG-001 might be considered a novel biomarker and therapeutic target for myocarditis [26]. Thus, these findings provide some evidence for the association between aberrant tsRNAs expression and cardiovascular system dysfunction.

This study was conducted to determine the expression profiles of tsRNAs in PCH patients by RNA sequencing, and the results were validated by real-time qPCR in larger groups and Ang II-stimulated H9c2 cells. We then analyzed the clinical diagnostic value of tsRNAs and investigated their possible biological function in the pathogenesis of PCH.

## 2. Materials and Methods

### 2.1. Clinical Samples Collection

Between August 2021 and July 2022, 60 subjects aged 40–80 years (35 PCH patients and 25 healthy volunteers as controls) were enrolled in the study. The PCH patients were recruited from the Department of Cardiology of The Affiliated Suzhou Hospital of Nanjing Medical University, Gusu School (Suzhou, China), and the healthy volunteers were recruited from the Suzhou Physical Examination Center (Suzhou, China). The inclusion criteria of the PCH patients were based on the “Chinese guidelines for the diagnosis and treatment of heart failure 2018”; patients with HFpEF and HFrEF were selected for inclusion. Patients with acute infection, myocardial infarction, severe primary disease, cerebral infarction, trauma, pregnancy, cancer, and drug or alcohol addiction were excluded from the study. During the same period, 25 healthy volunteers were recruited as the controls. The study was approved and supervised by the Ethics Committees of Affiliated Suzhou Hospital of Nanjing Medical University, Gusu School (K-2021-GSKY20210202).

### 2.2. Collection and Preparation of Plasma Samples

A total of 5 mL of blood was taken using EDTA vacuum anticoagulant blood collection tubes from the two groups. All participants fasted overnight before blood collection. After centrifuging the blood samples at 1000× *g* for 10 min (4 °C), the plasma samples were collected and stored at −80 °C.

### 2.3. Library Preparation and tRFs and tiRNAs Sequencing

Plasma samples from four healthy volunteers were numbered A1 to A4 as the controls, and those from four PCH patients were numbered B1 to B4. The total RNA was extracted with TRIzol following the manufacturer’s instructions. tsRNAs are heavily decorated with RNA modifications, which makes it difficult to construct small-RNA sequence libraries. Before library preparation for total RNA samples, some treatments were performed as follows: to ligate the 3′ adaptor, 3′ aminoacyl (charged) was deacylated to 3′-OH and 3′-cP (2′, 3′-cyclic phosphate) was removed to generate 3′-OH. To ligate the 5’-adaptor, 5′-OH (hydroxyl group) was phosphorylated to form 5’-P. In addition, m1A and m3C were demethylated. To sequence the RNA biotypes, sequencing libraries were size-selected using automated gel cutters. Agilent BioAnalyzer 2100 was used to qualify and quantify the libraries. The tsRNAs sequencing was performed by Shanghai Kangcheng Technology Co., Ltd., Shanghai, China.

### 2.4. Data Analysis of tsRNAs

Raw sequencing data generated from an Illumina NextSeq 500 that passed the Illumina chastity filter were used for the following analysis. Aligned trimmed reads (trimmed 5′, 3′-adaptor bases) were using bowtie 2 software (2.4.4) to align. Their abundance was assessed by the sequencing counts of tsRNAs and normalized as counts per million of total aligned reads (CPM). The differentially expressed tsRNAs were screened based on the count value with R package edgeR. Principal component analysis (PCA), pie plots, hierarchical clustering, scatter plots, and volcano plots were calculated and graphed in an R or Perl environment.

### 2.5. Quantitative Real-Time PCR Validation

Real-time qPCR was used to validate the expression changes detected by RNA-sequencing of tsRNAs in a larger sample size and in Ang II-stimulated H9c2 cells. There were four upregulated tsRNAs, including tRF-19-8FFDXXE5, tRF-19-SWRYVMH0, tRF-16-Q622EVE, and tRF-26-YONONU3INDD, and five downregulated tsRNAs, including tRF-21-NB8PLML3E, tRF-18-F9LKXN05, tRF-21-SWRYVMMV0, tRF-16-R29P4PE, and tRF-30-3JVIJMRPFQ5D. We validated the five downregulated tsRNAs. A miRNA of *C. elegans*, cel-miR-39-3p, was artificially added to the plasma as an external control (5 μL, 200 nM), and U6 served as an internal reference for the cell model. The total RNA was extracted from the plasma with TRIzol reagent (Thermo Fisher Scientific, Inc., Waltham, MA, USA), and 250 μL plasma of each subject was used. Then, the RNA was reverse-transcribed into cDNA and quantified by qPCR using PrimeScript™ RT Master Mix and TB Green^®^ Premix Ex Taq™ II (Takara, Inc., San Jose, CA, USA) in accordance with the manufacturer’s protocols. Reverse transcription was performed in 20 μL reaction volume, including 4 μL 5xRT Mix, 1.2 μL tRF RT primer (1 μM), 6 μL RNA, and 8.8 μL RNase free H_2_O. The reaction was performed at 37 °C for 15 min, followed at 85 °C for 5 s. The primers used in this experiment are shown in Table S1. PCR was performed in 20 μL reaction volume, including 10 μL of 2× Real-time PCR Master Mix, 0.8 μL tRFs & tiRNA specific Primer set (10 μM), 2 μL cDNA, and 7.2 μL RNase-free H2O. The reaction was performed at 95 °C for 30 s, followed by 40 amplification cycles at 95 °C for 5 s, and 60 °C for 30 s. The relative tsRNAs expression levels were calculated using the 2^−ΔΔCt^ method and were normalized to cel-miR-39-3p and U6.

### 2.6. Cell Culture and Treatment

The rat H9c2 cell line was obtained from Fuheng BioLogy (Shanghai, China), cultured in DMEM medium supplemented with 10% fetal bovine serum, 100 IU/mL penicillin, and 100 μg/mL streptomycin and kept in an incubator containing 5% CO_2_ at 37 °C in a humidified atmosphere. After H9c2 cells were grown to 70% confluence, they were treated with 2 mM Ang II for 24 h. Finally, the cultured cells were collected and the expression of tRF-30-3JVIJMRPFQ5D, tRF-16-R29P4PE, tRF-21-NB8PLML3E, and tRF-21-SWRYVMMV0 was detected.

After the cells were stimulated with Ang II for 24 h, tRF-21-NB8PLML3E mimic or inhibitor (GenePharma, Inc., Sunnyvale, CA, USA) was transfected for another 24 h, respectively. Transfection was conducted according to the manufacturer’s instructions. In brief, 5 μL transfection reagent, 150 pmol mimic, inhibitor, or NC were, respectively, diluted in 50 μL serum-free medium, then mixed and kept for 15 min at room temperature to prepare the transfection solution. After treatment of the H9c2 cells with the transfection solution for 4 h, the cells were cultured in a fresh medium containing 10% fetal bovine serum for an additional 20 h. After that, atrial natriuretic peptide (ANP) and brain natriuretic peptide (BNP) in the cell supernatant were detected using an ELISA kit (Sinobestbio, Inc., Xingtai, China) in accordance with the manufacturer’s instructions.

### 2.7. Fluorescence Staining

Fluorescence staining was used to observe tRF-21-NB8PLML3E mimic or inhibitor (GenePharma, Inc.)-treated Ang II-stimulated H9c2 cells. The cells were fixed with 4% paraformaldehyde for 30 min, then incubated with 0.1% Triton X-100 for 30 min, and, finally, blocked with 1% BSA for 1 h at room temperature. Phalloidin was placed on the glass slides and incubated for 20 min. DAPI was used to stain the nuclei. A confocal microscope was used to photograph the cells.

### 2.8. Bioinformatic Analysis

To analyze the possible biological functions of the differentially expressed tsRNAs, the target genes of the tsRNAs were predicted using miRanda and TargetScan, and the Gene Ontology (GO) project and the KEGG database were used to analyze the biological functions of the predicted target genes.

### 2.9. Statistical Analysis

The results were reported as the mean ± standard deviation (SD). Student’s *t* test was used for two-group comparisons of quantitative data, and the χ^2^ test was performed for two-group comparisons of nonquantitative data. The clinical diagnostic value of the candidate tsRNAs was evaluated by receiver operating characteristic (ROC) curve analysis, and the linear correlation in different groups was determined by Spearman analysis. *p* values < 0.05 were considered significant. All statistical analyses were performed using SPSS 19.0 (SPSS, Chicago, IL, USA) and GraphPad Prism 8 (GraphPad, San Diego, CA, USA).

## 3. Results

### 3.1. Characteristics of PCH Patients and Healthy Subjects

A total of 35 PCH patients and 25 healthy volunteers were enrolled in the experiment, and their baseline characteristics are shown in Table S2. The majority of PCH patients were male. A total of 37% of PCH patients had diabetes mellitus and 80% had hypertension, which was significantly higher than in the control group.

We divided the 35 patients with PCH into two groups based on echocardiography, including 16 cases of concentric hypertrophy and 19 cases of eccentric hypertrophy. The cardiac structure and function of the two types of patients are shown in Table 1. According to the results of echocardiography, the left ventricular end-diastolic diameter (LVEDD), left ventricular end-systolic diameter (LVESD), and right ventricular diameter (RV) in the patients with concentric hypertrophy were significantly lower than those in the patients with eccentric hypertrophy; in contrast, the thickness of the interventricular septum (IVS), left ventricular posterior wall dimensions (LVPWd) and left ventricular ejection fraction (LVEF) were significantly higher than those in the patients with eccentric hypertrophy.

### 3.2. Overview Expression of tsRNAs in PCH and Control Groups

**Figure 2:**
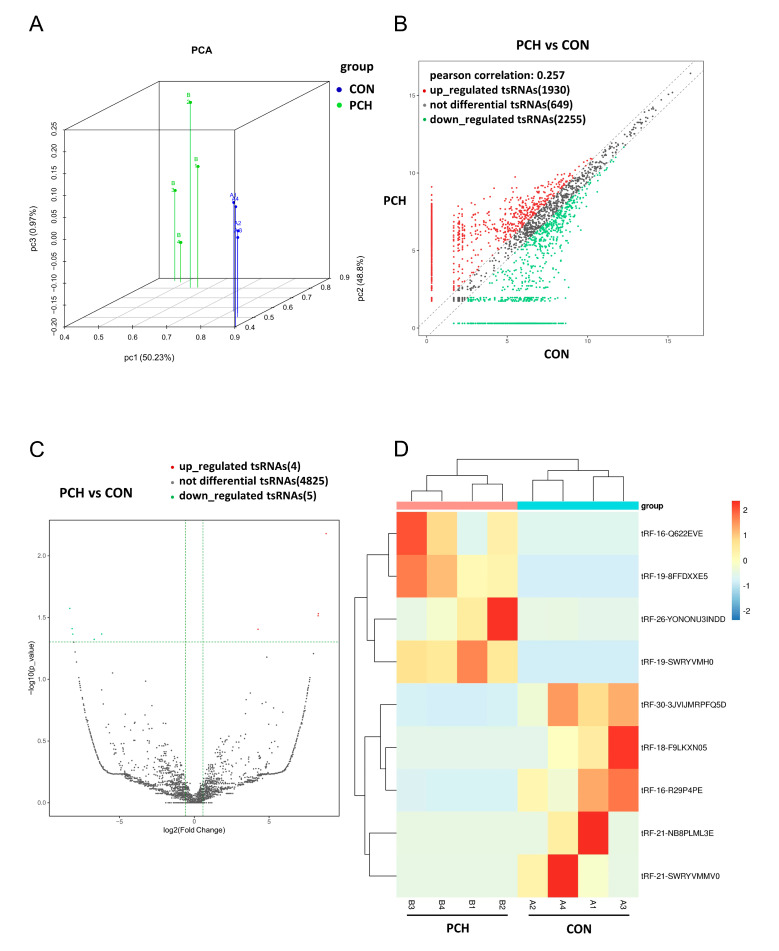
Expression profiles of tsRNAs sequencing data in the plasma of PCH patients and controls. (**A**) Primary component analysis. The X, Y, and Z axes represent the three main factors that affected the expression level of the samples. The colored point represents the corresponding samples, and the location of it shows the main character of the samples. Space distance represents the similarity of data size. (**B**) The scatter plot between two groups for tsRNAs. The CPM values of all tsRNAs are the plot. The values of the X and Y axes in the scatter plot are the averaged CPM values of each group (log2 scaled). tsRNAs above the top line (red dots, upregulation) or below the bottom line (green dots, downregulation) indicate more than a 1.5-fold change between the two compared groups. Gray dots indicate nondifferentially expressed tsRNAs. (**C**) The volcano plot of tsRNAs. The values of the X and Y axes in the volcano plot are log2 transformed fold change and −log10 transformed *p*-values between the two groups, respectively. Red/green circles indicate statistically significant differentially expressed tsRNAs with fold change no less than 0.5 and *p*-value ≤ 0.05 (Red: upregulated; Green: downregulated). Gray circles indicate nondifferentially expressed tsRNAs, with FC and/or q-value not meeting the cutoff thresholds. (**D**) Heatmap showing the hierarchical clustering of differentially expressed tsRNAs. CON: control; PCH: pathological cardiac hypertrophy.

To identify the expression levels of tsRNAs in the PCH and control groups, tsRNA sequencing was used for analysis. After quality-filtering of the library data, we used PCA for dimensionality reduction and visual presentation of large data sets; that is, tsRNAs with an ANOVA *p*-value ≤ 0.05 on counts per million (CPM) value were used to show the distinguishable tsRNAs expression profiling among the samples (Figure 2A).

Differentially expressed tsRNA analysis was performed with the R package edgeR and visualized with scatter plots. Fold change (>1.5) was used for screening the differentially expressed tsRNAs. As shown in Figure 2B, compared with the control group, 1930 tsRNAs were upregulated, 2255 tsRNAs were downregulated, and 649 tsRNAs were not observed to be differentially expressed.

A volcano plot is also a kind of scatter map. A volcano plot was used to quickly and intuitively identify tsRNAs with a large variation amplitude and statistical significance. The horizontal axis is Log2 (fold change) of tsRNA expression between the two groups, used to represent the multiple of difference. The vertical axis is −Log 10 (adjusted *p*-value) to show the significance of the difference. As shown in Figure 2C, four tsRNAs were significantly upregulated and five tsRNAs were significantly downregulated, suggesting that tsRNAs might have potential biological functions in PCH.

The results from hierarchical clustering shown by heat map revealed the differences in the tsRNAs expression profiles among the samples. Compared with the control group, the expression of nine tsRNAs in the PCH group was significantly dysregulated (Figure 2D).

### 3.3. Validation for the Differentially Dysregulated Expression of tsRNAs

**Figure 3:**
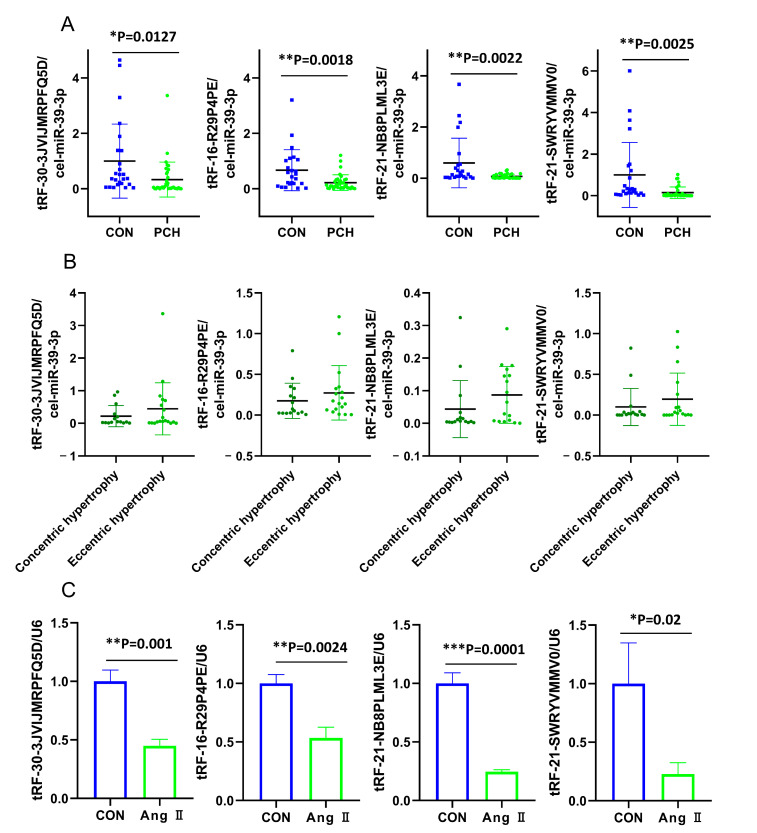
Validation of the relative expression level of downregulated tsRNAs by real-time qPCR. The plasma of 35 PCH patients and 25 controls were used for validation. The statistical significance between PCH and controls was calculated by the Student *t*-test. Data were expressed as the means ± SD. (**A**) The relative expression of the 4 down-regulated tsRNAs between PCH and CON. The blue dots mean expressions of CON, the green dots mean expressions of PCH. (**B**) The relative expression of the 4 downregulated tsRNAs between concentric hypertrophy and eccentric hypertrophy. *p* < 0.05 was considered statistically significant. The dark green dots mean expressions of CON, the light green dots mean expressions of PCH. (**C**) The relative expression of 4 down-regulated tsRNAs between the control H9c2 group and the Ang II-stimulated H9c2 group (*n* = 3). *p* < 0.05 was considered statistically significant. * *p* < 0.05, ** *p* < 0.01, *** *p* < 0.001. CON: control; PCH: pathological cardiac hypertrophy.

Real-time qPCR was performed to validate the five downregulated tsRNAs. tRF-18-F9LKXN05 could not be detected due to low expression levels in the plasma. As shown in Figure 3A, the expression levels of tRF-30-3JVIJMRPFQ5D, tRF-16-R29P4PE, tRF-21-NB8PLML3E, and tRF-21-SWRYVMMV0 were significantly downregulated (*p* < 0.05 or *p* < 0.01). With the progression of PCH, the expression levels of all four downregulated tsRNAs seemed to increase in the eccentric hypertrophy stage compared to the concentric hypertrophy stage but the differences were not significant (Figure 3B). They were notably decreased in the Ang II-stimulated H9c2 cells (Figure 3C), which was consistent with changes in the plasma. To verify that the five downregulated tsRNAs are not platelet-derived and to confirm the effects of age on the results, we re-recruited five PCH patients (with an average age of 65) and five healthy volunteers (with an average age of 65). Two tubes of blood (5 mL/tube) were collected from each individual in each group and centrifuged 1000× *g* for 10 min and 5000× *g* for 20 min, respectively. As the results showed, there was no significant difference in the expression of tRF-30-3JVIJMRPFQ5D, tRF-16-R29P4PE, tRF-21-NB8PLML3E, and tRF-21-SWRYVMMV0 between the two centrifugation methods (Figure S1). The expressions of tRF-30-3JVIJMRPFQ5D, tRF-16-R29P4PE, tRF-21-NB8PLML3E, and tRF-21-SWRYVMMV0 were significantly downregulated (Figure S2) which suggested that in the present study, the age has little impact on the plasma tsRNAs levels.

### 3.4. ROC Analysis of Validated tsRNAs in PCH Patients

**Figure 4:**
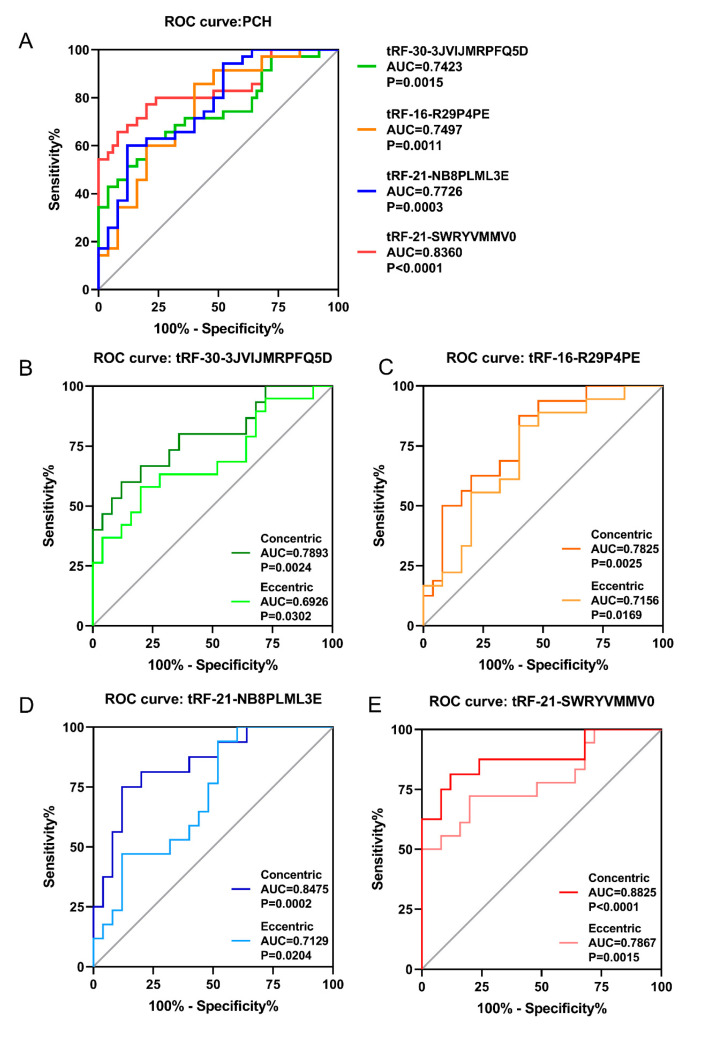
ROC curve analysis to evaluate the diagnostic value of validated tsRNAs. (**A**) ROC curves of the 4 downregulated tsRNAs in PCH (*n* = 35). (**B**–**E**) ROC curves of the 4 tsRNAs in concentric hypertrophy (*n* = 16) and eccentric hypertrophy (*n* = 19), respectively. AUC = area under curve; PCH: pathological cardiac hypertrophy; ROC = receiver operating characteristic.

According to the relative expression levels of the four proven tsRNAs (tRF-30-3JVIJMRPFQ5D, tRF-16-R29P4PE, tRF-21-NB8PLML3E, and tRF-21-SWRYVMMV0) in the plasma of PCH patients and healthy controls, ROC curves were generated to analyze the diagnostic potential of the differentially expressed tsRNAs (Figure 4A). The values of the area under the ROC curve (AUC) of tRF-30-3JVIJMRPFQ5D, tRF-16-R29P4PE, tRF-21-NB8PLML3E, and tRF-21-SWRYVMMV0 were 0.7423, 0.7497, 0.7726, and 0.836, respectively. A larger AUC generally means better diagnostic potential. In addition, considering the *p*-value, sensitivity, and specificity, tRF-21-SWRYVMMV0 and tRF-21-NB8PLML3E may be the preferred biomarkers for the diagnosis of PCH.

To analyze the early-stage screening potential of the differentially expressed tsRNAs in PCH, ROC curves were generated for the four tsRNAs in concentric hypertrophy and eccentric hypertrophy (Figure 4B–E), and the AUC, *p*-value, sensitivity, and specificity values were analyzed (Table S3). The ROC curve results showed that the early-stage screening potential of the four tsRNAs was better than that of the late stage, and tRF-21-SWRYVMMV0 and tRF-21-NB8PLML3E showed good diagnostic potential in the entire course of PCH.

### 3.5. Correlations of Validated tsRNAs Expression with Disease Phenotypes in PCH Patients

**Figure 5:**
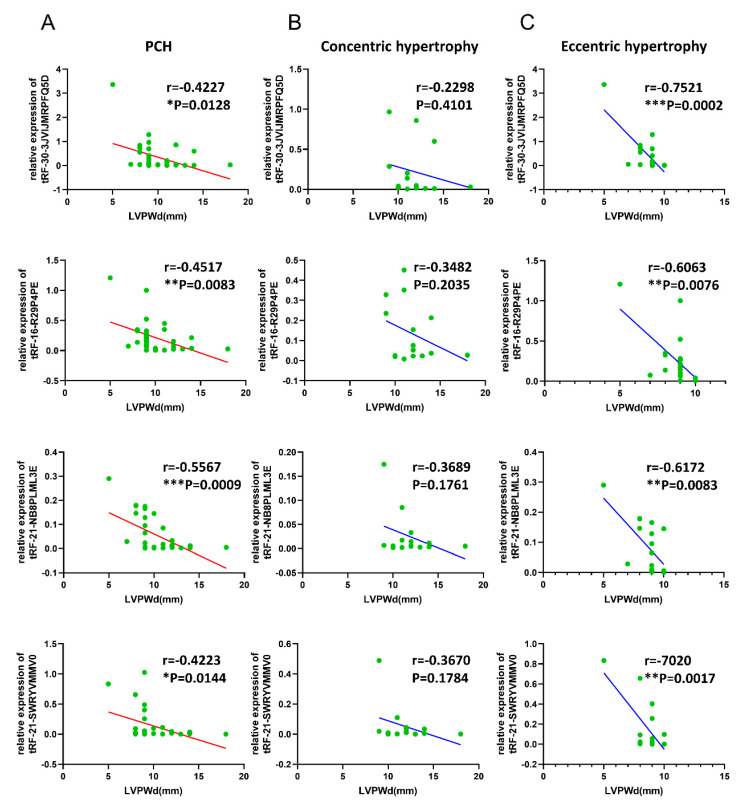
Correlations of validated tsRNAs expression with LVPWd in PCH patients. Spearman’s analysis was applied to test the correlation of validated tsRNAs expression with disease activity. (**A**) Correlations of the 4 downregulated tsRNAs expression with LVPWd in PCH (*n* = 35); (**B**): Correlations of the four downregulated tsRNAs expressions with LVPWd in concentric hypertrophy (*n* = 16); (**C**): Correlations of the four downregulated tsRNAs expressions with LVPWd in eccentric hypertrophy (*n* = 19). *p* < 0.05 was considered statistically significant. * *p* < 0.05, ** *p* < 0.01, *** *p* < 0.001. LVPWd: left ventricular posterior wall dimensions. PCH: pathological cardiac hypertrophy.

The disease phenotypes of PCH against the corresponding expression levels of the validated tsRNAs are listed in Table S4. The main pathological indicators of PCH are IVS, LVPWd, LVEDD, and LVEF. All four validated tsRNAs were significantly negatively correlated with LVPWd (Figure 5A) and were notably significantly negatively correlated with eccentric hypertrophy (Figure 5B,C). In addition, tRF-21-NB8PLML3E was significantly negatively correlated with LVEF (Table S4).

### 3.6. Effects of tRF-21-NB8PLML3E in Ang II-stimulated H9c2 Cells

**Figure 6:**
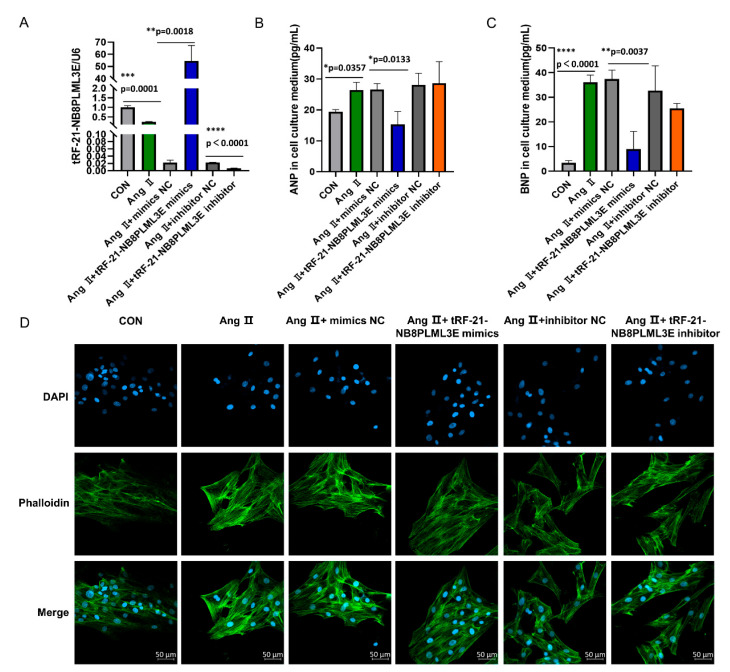
Intracellular tRF-21-NB8PLML3E, ANP, and BNP expressions as well as cytoskeleton staining in tRF-21-NB8PLML3E mimic/inhibitor-transfected Ang II-stimulated H9c2 cells. (**A**) tRF-21-NB8PLML3E expression. (**B**) ANP expression. (**C**) BNP expression. (**D**) immunofluorescence photo (phalloidin stained green, scale bar: 50 μm). Data are expressed as the mean ± SD, with *n* = 3 per group. *p* < 0.05 was considered statistically significant. * *p* < 0.05, ** *p* < 0.01, *** *p* < 0.001, **** *p* < 0.0001. CON: control; NC: Negative control.

The results showed that tRF-21-NB8PLML3E expression in the mimic- or inhibitor-transfected group was significantly higher and lower, respectively than that in the control group (Figure 6A, *p* < 0.01), indicating an effective transfection. As shown in Figure 6B,C, the levels of ANP and BNP were significantly decreased in tRF-21-NB8PLML3E mimic-transfected cells. The fluorescence results (Figure 6D) showed that the tRF-21-NB8PLML3E mimic treatment was able to alleviate Ang II-induced cardiomyocyte hypertrophy.

### 3.7. Biological Information Function Analysis of tsRNAs

**Figure 7:**
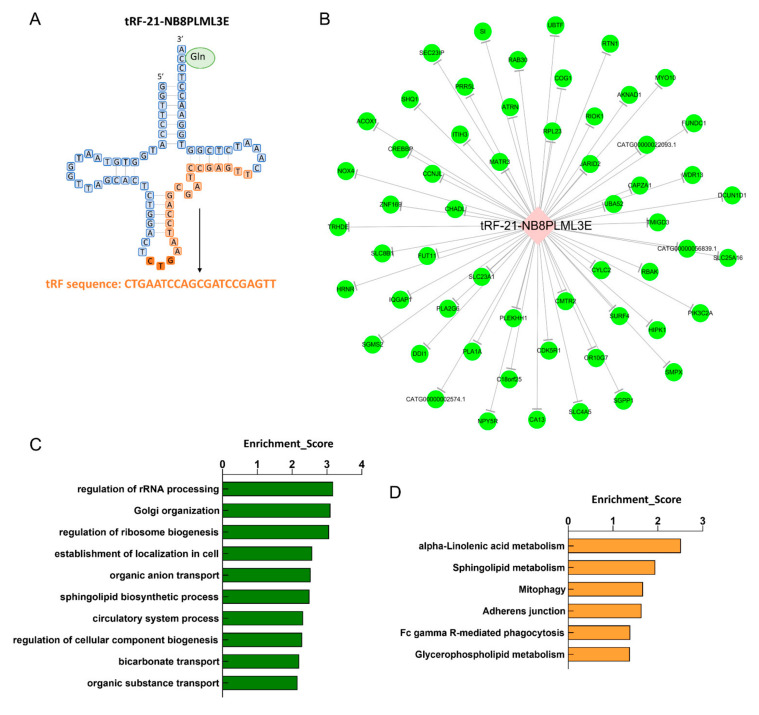
Integrated analysis of validated tsRNAs. (**A**) Sequence information for tRF-21-NB8PLML3E. (**B**) The potentially altered target mRNAs of tRF-21-NB8PLML3E. (**C**) The gene ontology (GO) enrichment analyses of tRF-21-NB8PLML3E. (**D**) KEGG pathway analysis for tRF-21-NB8PLML3E gathering genes. The vertical axis shows the annotated functions of the target genes. The horizontal axis shows the enrichment score (−log10 transformed *p*-value), respectively. Only the most significantly enriched clusters are included.

Figure 7A shows the sequence information of tRF-21-NB8PLML3E. Previous studies have shown that many tsRNAs can perform roles similar to miRNAs, including interacting with Argonaute, producing in a dicer-dependent manner, and participating in translation silencing [27,28]. Thus, to further explore the potential roles of tRF-21-NB8PLML3E in PCH, we identified the associated target genes with the miRNA target prediction algorithm TargetScan and the miRanda algorithm. The number of target genes was 56 for tRF-21-NB8PLML3E (Figure 7B).

To predict the potential biological functions of these target genes, we evaluated them using GO and KEGG enrichment analyses. In the biological process of the GO analysis, the top-10 enriched terms that may be related to cardiovascular disease are displayed in Figure 7C, indicating that the potential target genes of tRF-21-NB8PLML3E are mainly responsible for the biogenesis process of the ribosome and Golgi.

The significant pathways for each tsRNA were ranked by adjusted *p*-value according to the KEGG pathway analysis. The signaling pathway of tRF-21-NB8PLML3E was mainly enriched in alpha-linolenic acid metabolism, sphingolipid metabolism, mitophagy, adherens junction, Fc gamma R-mediated phagocytosis, and glycerophospholipid metabolism (Figure 7D).

## 4. Discussion

Given the pleiotropic roles of tsRNAs in the cardiovascular system, the purpose of this study was to investigate whether tsRNAs are involved in PCH. We first detected the expression profile of tsRNAs in the plasma of PCH patients and healthy volunteers by RNA sequencing. The results showed that nine tsRNAs were significantly differentially expressed. Then, we performed real-time qPCR to further validate the significantly downregulated tsRNAs in line with the sequencing data. As the results showed, the expression of tRF-30-3JVIJMRPFQ5D, tRF-16-R29P4PE, tRF-21-NB8PLML3E, and tRF-21-SWRYVMMV0 was significantly downregulated. However, with the progression of PCH, the expression levels of the four tsRNAs seemed to rebound slightly, which deserves further investigation. A ROC curve analysis among the four tsRNAs indicated that tRF-21-NB8PLML3E might serve as a biomarker for the early screening of PCH. Especially, in the present study, we observed that in Ang II-stimulated H9c2 cells, the expression of tRF-21-NB8PLML3E was decreased, and the addition of tRF-21-NB8PLML3E mimic simultaneously reversed the expression of tRF-21-NB8PLML3E, inhibited ANP and BNP expression, and attenuated cardiomyocyte hypertrophy. As the results showed, transfection with mimic control or inhibitor control significantly inhibited tRF-21-NB8PLML3E expression but resulted in virtually no changes in ANP and BNP levels. From this, we deduced that the changes in ANP and BNP levels were caused by Ang II. The decrease of tRF-21-NB8PLML3E could not further cause the changes in ANP and BNP levels, while the increase of tRF-21-NB8PLML3E would exert the biological activity and lead to the changes in ANP and BNP levels. From these experimental and literature data, we thought that tRF-21-NB8PLML3E could improve Ang II-induced H9c2 cells hypertrophy, but the specific mechanisms of tRF-21-NB8PLML3E effects on PCH need to be investigated.

Interestingly, through correlation analysis with clinical phenotypes, we found that the four tsRNAs showed negative correlations with LVPWd, and the correlation gradually increased with the progression of PCH. This result suggested that tsRNAs are more and more closely involved in the development of PCH, which also indicates that tsRNAs not only have the potential of biomarkers in the early screening and diagnosis of PCH but their potential effects in the treatment of PCH are also worthy of attention. Therefore, it is necessary to further explore their roles in pathogenesis.

To further confirm the roles of tRF-21-NB8PLML3E in PCH, the target genes were predicted by miRnada algorithms and TargetScan, and GO analysis and KEGG pathway analysis were performed on the target genes. As for tRF-21-NB8PLML3E, its target genes may be closely related to the biogenesis process of the ribosome as revealed by GO analysis. Supporting the result of this analysis, recent evidence has shown that stress-induced tsRNAs can flexibly promote or inhibit protein translation, which might be closely related to where and how they interact with the ribosome [20]. According to the KEGG pathway analysis, the six signaling pathways enriched by tRF-21-NB8PLML3E are mainly related to metabolism. It is generally accepted that a disorder of the cardiac metabolic process is the common pathogenesis factor of PCH [29,30]. It has been proved that impaired adaptation of energy metabolism can exacerbate pathological hypertrophy and increase cardiomyocyte death [31]. An impairment of fatty acid and pyruvate metabolism causes mitochondrial dysfunction, oxidative stress, and contractile dysfunction [32]. Dysregulation of fatty acid or carbohydrate metabolism directly induces cardiac hypertrophy and/or functional decline in genetically engineered mice by regulating pathological signaling mechanisms [33,34,35]. Therefore, we deduced that tRF-21-NB8PLML3E might exert regulatory effects on PCH, and the mechanism may partially involve the regulation of metabolic processes in the dysfunctional heart.

### Study Limitations

There are certain limitations to our study. First, the insufficient number of subjects may have led to some data bias. Second, in addition to playing roles similar to miRNAs, tsRNAs have other mechanisms of action. However, since there is no independent target-prediction algorithm for tsRNA at present, we chose the miRNA target-prediction algorithm for preliminary functional analysis to speculate the possible role of tsRNAs in PCH without experimental verification. Lastly, the elevated tsRNAs were not further characterized in the present study.

## 5. Conclusions

Our study provided the expression profiles of tsRNAs in the plasma of PCH patients for the first time. The validation results by integrated next-generation sequencing analysis and qPCR demonstrated that tsRNAs in plasma are valuable for the diagnosis of PCH. Furthermore, tRF-21-NB8PLML3E might be the preferred diagnostic biomarker for PCH. Nonetheless, further studies are required to explore the molecular mechanisms of tRF-21-NB8PLML3E in PCH development.

## Figures and Tables

**Table 1:** Cardiac structure and function in different stages of PCH.

Characteristics		Sequencing Samples	All Clinical Samples Collected		
		PCH (Average, *n* = 4)	Concentric Hypertrophy (*n* = 12–16)	Eccentric Hypertrophy (*n* = 11–19)	*p* Value
LV structure and function					
	LVEDD (mm)	45.5	49.375 ± 9.6116	61.9474 ± 7.9055	0.0002
	LVESD (mm)	31	36.375 ± 10.6701	51.5263 ± 8.8404	0.0001
	IVS (mm)	15.5	14.325 ± 3.9408	8.3 ± 1.6496	<0.0001
	LVPWd (mm)	13	12 ± 2.2804	8.6316 ± 1.1648	<0.0001
	LVEF (%)	57.5	50.5625 ± 12.0884	35.2632 ± 8.6207	0.0001
RV structure and function					
	RV diameter (mm)	29.25	31.1538 ± 4.2787	38.875 ± 8.123	0.0032
	TAPSE (mm)	19.25	20.3917 ± 6.1278	16.4786 ± 2.121	0.0547
	TAPSV (cm/s)	12.475	16.0417 ± 5.979	15.0714 ± 3.1146	0.6195
Diastolic function					
	Mitral E wave (cm/s)	3.1222	4.2258 ± 2.7983	3.3364 ± 2.2869	0.4159
	Mitral annular e′(cm/s)	4.425	5.0083 ± 1.9365	5.2182 ± 1.4084	0.7711
	E/e′ ratio	13	17.6333 ± 8.9467	15.5 ± 5.9564	0.4989
	E/A ratio	0.6875	1.2875 ± 0.5633	1.4458 ± 0.9514	0.6248
	TR velocity (m/s)	220	217.5 ± 51.93	266.3571 ± 52.9639	0.0351
heart failure marker					
	BNP (pg/mL)	80.5	433.8 ± 461.3022 (*n* = 5)	950.9167 ± 727.2138 (*n* = 12)	0.1657
	NT-proBNP (pg/mL)	/	3236.9231 ± 4410.0705 (*n* = 13)	9525.6667 ± 9690.4966 (*n* = 9)	0.0977

A: Mitral A wave, late diastolic mitral inflow velocity; BNP: B type natriuretic peptide; e′: early diastolic mitral annular tissue velocity; E: Mitral E wave, early diastolic mitral inflow velocity; IVS: the thickness of the interventricular septum; LVEDD: left ventricular end-diastolic diameter; LVESD: left ventricular end-systolic diameter; LVPWd: left ventricular posterior wall dimensions; LVEF: left ventricular ejection fraction; NT-proBNP: N terminal pro-B-type natriuretic peptide; PCH: pathological cardiac hypertrophy; RV: right ventricular; TAPSE: tricuspid annular plane systolic excursion; TAPSV: tricuspid annular peak systolic velocity; TR, tricuspid regurgitation. Data are mean ± standard deviation (SD). *p* < 0.05 was regarded as significant. the χ^2^ test and unpaired *t* test were used.

## Data Availability

The data that support the findings of this study are available from the corresponding author upon reasonable request.

## Supplementary Materials

The following supporting information can be downloaded at: https://www.mdpi.com/article/10.3390/diagnostics13122065/s1, Figure S1: The relative expression of the 4 down-regulated tsRNAs of 5 PCH patients and 5 healthy volunteers. Two plasma samples were collected in each group. 1: centrifuged 1000× *g* for 10 min; 2: centrifuged 5000× *g* for 20 min. *p* < 0.05 was considered statistically significant; Figure S2: The relative expression of the 4 down-regulated tsRNAs of 5 PCH patients (with the average age 65) and 5 healthy volunteers (with the average age 65). *p* < 0.05 was considered statistically significant; Table S1: The primer sequences; Table S2: Baseline characteristics; Table S3: ROC curve of significantly down-regulated tsRNA of PCH; Table S4: Pearson correlation coefficients of significantly decreased tsRNA and clinical parameters of PCH.
