# Supplementary material for: Global Profile of tRNA-Derived Small RNAs in Pathological Cardiac Hypertrophy Plasma and Identification of tRF-21-NB8PLML3E as a New Hypertrophy Marker
Source: Diagnostics (Basel). 2023 Jun 14;13(12):2065. doi: 10.3390/diagnostics13122065 (PMC10297010; doi:10.3390/diagnostics13122065)
Supplement: Supplementary file 1 [file diagnostics-13-02065-s001.zip › Figure S1 and S2 captions.pdf]

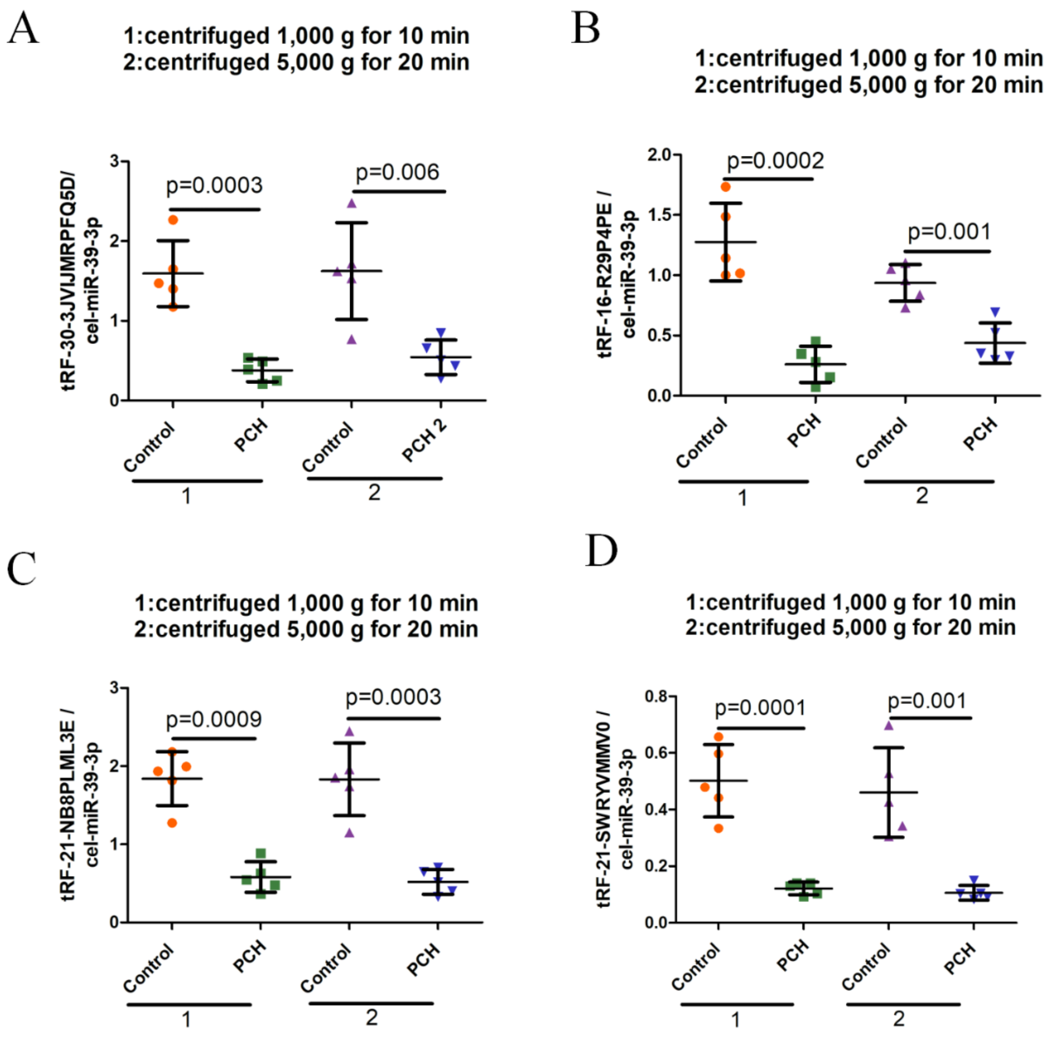


**Figure S1** The relative expression of the 4 down-regulated tsRNAs of 5 PCH patients and 5 healthy volunteers. Two plasma samples were collected in each group. 1: centrifuged 1,000 g for 10 min; 2: centrifuged 5,000 g for 20 min. $p<0.05$ was considered statistically significant.

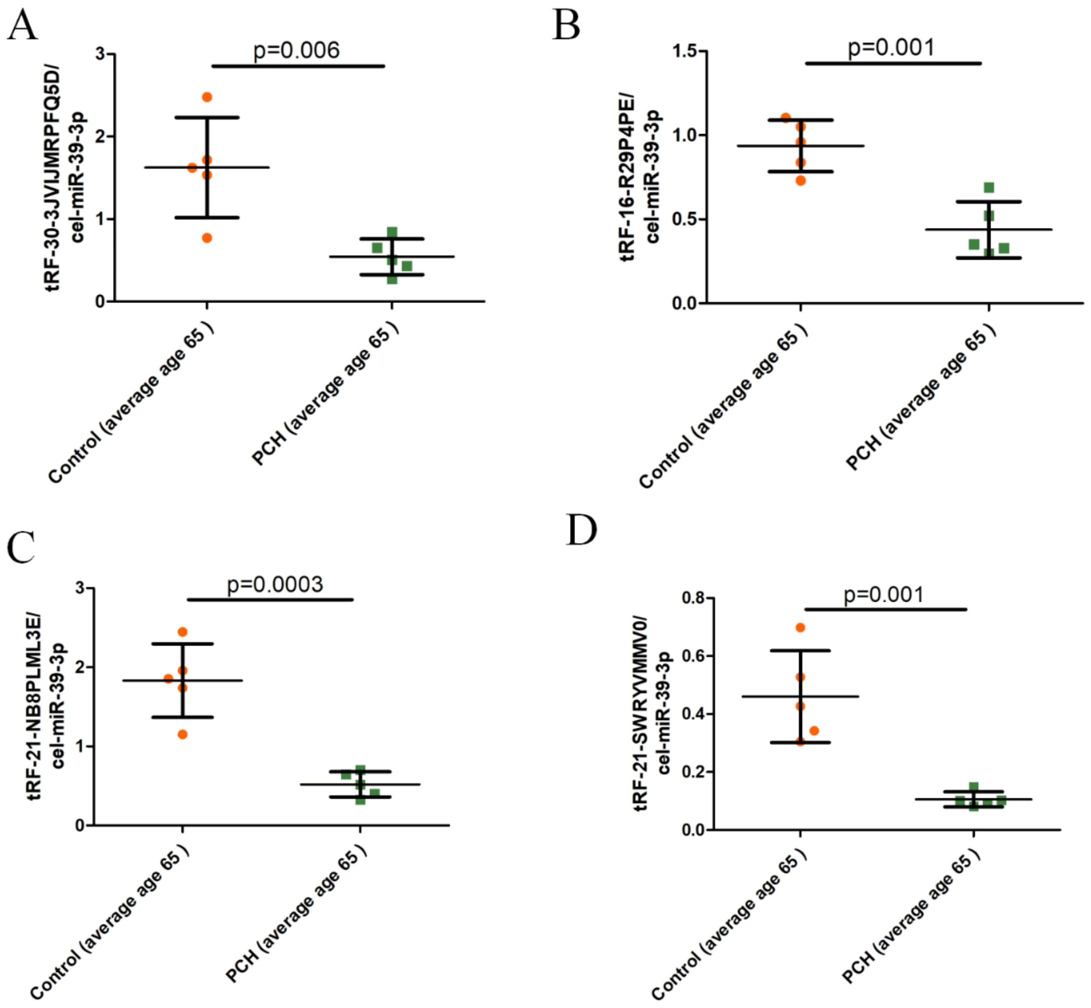


**Figure S2** The relative expression of the 4 down-regulated tsRNAs of 5 PCH patients (with the average age 65) and 5 healthy volunteers (with the average age 65). $p<0.05$ was considered statistically significant.
