# Supplementary material for: Global Profile of tRNA-Derived Small RNAs in Pathological Cardiac Hypertrophy Plasma and Identification of tRF-21-NB8PLML3E as a New Hypertrophy Marker
Source: Diagnostics (Basel). 2023 Jun 14;13(12):2065. doi: 10.3390/diagnostics13122065 (PMC10297010; doi:10.3390/diagnostics13122065)
Supplement: Supplementary file 1 [file diagnostics-13-02065-s001.zip › Supplementary_Material-Tables.pdf]

Table S1 The primer sequences.

ncRNA	Primer sequence
cel-miR-39-3p	non-disclosure agreement(ribobio,lnc.)
U6	5'-CGCTTCGGCACATATAC-3'
	5'-TTCACGAATTTGCGTGTCATC-3'
tRF-30-3JVIJMRPFQ5D	5'-CCGGCTAGCTCAGTCGGTAGA-3'
	5'-TATGGTTGTTCACGACTCCTTCAC-3'
tRF-16-R29P4PE	5'-CTCAGGTTGTGGGGATGTAGCT-3'
	5'-TATGGTTGTTCACGACTCCTTCAC-3'
tRF-21-NB8PLML3E	5'-CAGGTTGTCTGAATCCTTCAC-3'
	5'-TATGGTTGTTCACGACTCCTTCAC-3'
tRF-21-SWRYVMMV0	5'-GCTACGCGTCGTGGTTGTAGT-3'
	5'-TATGGTTGTTCACGACTCCTTCAC-3'

Table S2 Baseline characteristics.

characteristics	Sequencing samples		all clinical samples collected		
	CON (*n*=4)	PCH(*n*=4)	CON (*n*=25)	PCH(*n*=35)	*p* value
Age (years)	49.25	56	**50.08±7.5051**	**65.4±15.1758**	**<0.0001**
Female, *n* (%)	0 (0%)	0 (0%)	6 (24%)	9 (25.7%)	0.8798
Body surface area (m2)	1.786	1.8332	1.7776±0.147	1.7923±0.2319	0.7846
Body mass index (kg/m2)	23.5835	26.0524	24.302±3.11	25.9447±4.9706	0.1575
Smoking (%)	2 (50%)	4 (100%)	6 (24%)	14 (40%)	0.1949
Drinking (%)	3 (75%)	2 (50%)	6 (24%)	9 (25.7%)	0.8798
Co-morbidities					
Diabetes mellitus, *n* (%)	0 (0%)	4 (100%)	**1 (4%)**	**13 (37.1429%)**	**0.0028**
Hypertension, *n* (%)	0 (0%)	2 (50%)	**5 (20%)**	**28 (80%)**	**<0.0001**
Dyslipidaemia, *n* (%)	0 (0%)	1 (25%)	1 (4%)	5 (14.3%)	0.3827

CON: control; PCH: pathological cardiac hypertrophy

Table S3 ROC curve of significantly down-regulated tsRNA of PCH.

tsRNA	course of PCH	AUC	SEM	*p* value	95% CI	*Cutoff* value	Sensitivity%	Specificity%
tRF-30-3JVIJMRPFQ5D	PCH	0.7423	0.0628	0.0015	0.6192 to 0.8654	0.0423	42.86	96.00
	Concentric hypertrophy	0.7893	0.0772	0.0024	0.6381 to 0.9406	0.0409	46.67	96.00
	Eccentric hypertrophy	0.6926	0.0831	0.0302	0.5297 to 0.8555	0.0423	36.84	96.00
tRF-16-R29P4PE	PCH	0.7497	0.0652	0.0011	0.6219 to 0.8775	0.0228	17.14	96.00
	Concentric hypertrophy	0.7825	0.0717	0.0025	0.6419 to 0.9231	0.0539	50.00	92.00
	Eccentric hypertrophy	0.7156	0.0788	0.0169	0.5610 to 0.8701	0.0219	16.67	96.00
tRF-21-NB8PLML3E	PCH	0.7726	0.0614	0.0003	0.6522 to 0.8930	0.0049	25.71	96.00
	Concentric hypertrophy	0.8475	0.0621	0.0002	0.7258 to 0.9692	0.0049	37.50	96.00
	Eccentric hypertrophy	0.7129	0.0792	0.0204	0.5578 to 0.8681	0.0049	17.65	96.00
tRF-21-SWRYVMMV0	PCH	0.8360	0.0511	<0.0001	0.7359 to 0.9361	0.0324	57.14	96.00
	Concentric hypertrophy	0.8825	0.0603	<0.0001	0.7644 to 1.000	0.0308	62.50	96.00
	Eccentric hypertrophy	0.7867	0.0740	0.0015	0.6417 to 0.9317	0.0308	50.00	96.00

AUC: area under curve; CI: confidence interval; PCH: pathological cardiac hypertrophy; ROC: receiver operating characteristic; SEM: standard error of mean; tsRNA: tRNA-derived small RNAs. $p<0.05$ was regarded as significant.

Table S4 Pearson correlation coefficients of significantly decreased tsRNA and clinical parameters of PCH.

tsRNA	course of PCH	IVS (mm)		LVPWd (mm)		LVEDD (mm)		LVEF (%)	
		r value	*p* value	*r* value	*p* value	*r* value	*p* value	*r* value	*p* value
tRF-30-3JVIJMRPFQ5D	PCH	-0.0889	0.6172	**-0.4227**	**0.0128**	0.1151	0.5169	-0.2953	0.0900
	Concentric hypertrophy	-0.2991	0.2788	-0.2298	0.4101	0.3010	0.2756	-0.3234	0.2397
	Eccentric hypertrophy	0.3208	0.1805	**-0.7521**	**0.0002**	-0.0958	0.6965	-0.2598	0.2827
tRF-16-R29P4PE	PCH	-0.0459	0.7968	**-0.4517**	**0.0083**	0.0064	0.9712	-0.1777	0.3148
	Concentric hypertrophy	-0.0533	0.8447	-0.3482	0.2035	0.2663	0.3188	-0.3062	0.2487
	Eccentric hypertrophy	0.4104	0.0907	**-0.6063**	**0.0076**	-0.3874	0.1122	0.0587	0.8170
tRF-21-NB8PLML3E	PCH	-0.1906	0.2880	**-0.5567**	**0.0009**	0.3223	0.0674	**-0.4630**	**0.0067**
	Concentric hypertrophy	-0.0556	0.8381	-0.3689	0.1761	0.2821	0.2898	-0.4513	0.0793
	Eccentric hypertrophy	0.0787	0.7640	**-0.6172**	**0.0083**	0.1368	0.6006	-0.3712	0.1424
tRF-21-SWRYVMMV0	PCH	-0.1067	0.5482	**-0.4223**	**0.0144**	0.1975	0.2628	-0.3400	0.0613
	Concentric hypertrophy	-0.2424	0.3657	-0.3670	0.1784	0.3847	0.1412	-0.4589	0.0738
	Eccentric hypertrophy	0.4332	0.0725	**-0.7020**	**0.0017**	-0.0681	0.7885	-0.1578	0.5316

IVS: the thickness of the interventricular septum; LVEDD: left ventricular end-diastolic diameter; LVPWd: left ventricular posterior wall dimensions; LVEF: left ventricular ejection fraction; PCH: pathological cardiac hypertrophy. $p<0.05$ was regarded as significant.
